# The Inhibitory Effects of *Heterotrigona Itama* Honey Marinades on the Formation of Carcinogenic Heterocyclic Amines in Grilled Beef Satay

**DOI:** 10.3390/molecules25173874

**Published:** 2020-08-26

**Authors:** Sharina Shamsudin, Jinap Selamat, Maimunah Sanny, Nuzul Noorahya Jambari, Rashidah Sukor, Sarva Mangala Praveena, Alfi Khatib

**Affiliations:** 1 Faculty of Food Science and Technology, Universiti Putra Malaysia (UPM), Serdang 43400, Selangor, Malaysia; sharina@mardi.gov.my (S.S.); s_maimunah@upm.edu.my (M.S.); nuzuljambari@gmail.com (N.N.J.); rashidah@upm.edu.my (R.S.); 2 Food Science and Technology Research Centre, Malaysian Agricultural Research and Development Institute (MARDI), Persiaran MARDI-UPM, Serdang 43400, Selangor, Malaysia; 3 Food Safety and Food Integrity (FOSFI), Institute of Tropical Agriculture and Food Security, Universiti Putra Malaysia (UPM), Serdang 43400, Selangor, Malaysia; 4 Department of Environmental and Occupational Health, Faculty of Medicine and Health Sciences, Universiti Putra Malaysia (UPM), Serdang 43400, Selangor, Malaysia; smpraveena@upm.edu.my; 5 Pharmacognosy Research Group, Faculty of Pharmacy, International Islamic University Malaysia, Kuantan 25200, Pahang, Malaysia; alfikhatib1971@gmail.com

**Keywords:** heterocyclic amines (HCAs), stingless bee honey, grilled beef, botanical origin, antioxidant activity (IC_50_), partial least squares (PLS)

## Abstract

Heterocyclic amines (HCAs) are carcinogenic food toxicants formed in cooked meats, which may increase the risk of cancer development in humans. Therefore, in this study, the effect of stingless bee honey from different botanical origins on the formation of HCAs in grilled beef satay was investigated. HCAs concentration in grilled beef satay was determined by using high performance liquid chromatography (HPLC). In total, six of the most toxigenic HCAs representing aminoimidazo-azaarenes (AIAs) (MeIQx, 4,8-DiMeIQx, and PhIP) and amino carbolines (norharman, harman, and AαC) groups were identified in all the beef samples investigated. A significant reduction in HCAs was observed in grilled beef marinated in honey as compared to beef samples marinated in table sugar (control), in which the reduction of 95.14%, 88.45%, 85.65%, and 57.22% was observed in gelam, starfruit, acacia, and *Apis* honey marinades, respectively. According to the partial least squares regression (PLS) model, the inhibition of HCAs in grilled beef was shown to be significantly correlated to the antioxidant activity (IC_50_) of the honey samples. Therefore, the results of this study revealed that the addition of stingless bee honey could play an important role in reducing HCAs in grilled beef.

## 1. Introduction

The exposure to the high amounts of heterocyclic amines (HCAs) increases the risk of cancer in humans [1] as some compounds which occur in muscle food such as poultry, meat, and fish can become carcinogenic when cooked at temperatures above 150 °C [2]. The International Agency for Research on Cancer (IARC) has classified HCAs into two categories known as class 2A and class 2B based on their carcinogenicity effect toward humans [3]. To date, more than 25 HCAs have been identified in various cooked food items [4] and they are classified based on their formation process into two main groups, namely the aminoimidazo-azaarenes (AIAs) and amino carbolines. AIAs are commonly found in cooked meat at temperatures below 250 °C [5], while amino carbolines are formed at higher temperatures above 250 °C through the pyrolysis of proteins or amino acids [6]. The Maillard reaction has been reported to be involved in the generation of AIAs through complex chemical reactions between the amino acids, reducing sugars, and creatine or creatinine that is present in meat [5]. In general, the main cooking methods that lead to higher concentrations of HCAs in food are grilling and frying [2].

The formation of HCAs can be influenced by several factors such as the meat type, meat quality, cooking conditions, cooking materials, pH, fat level, heat and mass transfer, antioxidants, and precursor concentrations [7,8,9,10,11,12,13]. The differences in the types and concentrations of HCAs are considerably dependent on the cooking method and types of meat used [5]. In general, the level of HCAs in meat products varies from 0 to 500 ng/g [14], in which PhIP, MeIQx, 4,8-DiMeIQx, MeIQ, norharman, and harman were shown to be commonly detected in cooked meat [6]. Among the different types of HCAs, PhIP and MeIQx are the most abundant and commonly found in cooked meat [15]. On the other hand, AαC is normally detected in grilled, barbecued or high temperature-fried meat and fish [15].

Since the consumption of HCAs can increase the risk of cancer development in humans, the exposure to HCAs must be reduced as recommended by IARC. Nevertheless, the exposure to HCAs cannot be avoided as meat is the main protein source and cooked meat is typically consumed by humans. Several studies have indicated that the addition of antioxidants as one of the ingredients when cooking meat dishes can reduce the formation of HCAs to some extent according to the types and concentrations of antioxidants used [16]. Numerous studies have investigated the impact of antioxidants on the formation of HCAs using different sources of antioxidants such as plant extracts/tissues [17,18,19], spices [20], extra virgin olive oil [21], and synthetic antioxidants [12]. These studies have indicated that antioxidants have an inhibitory effect toward reducing the formation of HCAs based on experiments using a model system and cooked meat, in which they act as a scavenger of free radicals or metabolite compounds that are formed during the Maillard reaction and subsequently reduce the formation of HCAs [22,23].

Honey is a good source of antioxidants, in which it was previously reported that honey can reduce the HCAs content in cooked meats when used as one of the marinating ingredients prior to the cooking process [24]. The effect of honey from *Apis mellifera* [25,26] and *Apis dorsata* (Tualang honey) [27] were previously evaluated, in which low concentrations of HCAs were detected. To date, studies on the effect of honey-containing marinades are scarce and there is no published data on the effect of *Heterotrigona itama* honey (stingless bee honey) on the formation of HCAs in cooked meats. Thus, we hypothesized that the addition of stingless bee honey in marinade ingredients would be effective in reducing the HCAs formation during the cooking process. The objective of this study was to evaluate the effect of stingless bee honey-containing marinades on the formation of HCAs and to determine the factor that influence HCAs formation in grilled beef satay. 

## 2. Results and Discussion

### 2.1. Marinade Uptake

The marinade uptake of beef meat is shown in Table 1. The percentage of marinade uptake of beef meat marinated with table sugar showed the lowest value (26.21%) and was significantly different than beef meat marinated with stingless bee honey (31.12–33.32%) and honeybee honey (29.78%). The marinade uptake can potentially be affected by the pH of the marinade used during the marinating process. Since the honey samples used in this study had low pH values (3.00–3.56), it is, therefore, possible for the pH to have an effect on the marinade uptake of beef meat during the marinating process. Additionally, the pH of the marinade has been reported to influence the water holding capacity and water absorption of meat. For instance, a low pH marinade has a higher water holding capacity and water absorption because of the increased ionic strength and net negative charge produced in the meat [28]. Furthermore, among the different types of stingless bee honey marinades investigated, no significant differences were observed in the marinade uptake of these samples. These results are consistent with the findings by Yucel et al. [29], in which no significant difference was observed in the weight gain of breast meat marinated with different concentrations of honey. 

### 2.2. Cooking Loss

The percentage of cooking loss of the grilled beef samples ranged from 28.10% to 35.38% (Table 1). The cooking loss of grilled beef is linked to water loss during the grilling process. Unmarinated samples showed a significantly higher (35.38%) cooking loss as compared to other samples. However, the cooking loss of grilled beef marinated with stingless bee honey ranged from 28.10% to 28.88% and was significantly lower than unmarinated samples and beef samples marinated with honeybee honey. Unmarinated beef had the highest cooking loss and this observation may be attributed to the direct contact with heat and induced water loss from the meat [27,30]. However, the variations in cooking loss observed for beef meat marinated with honeybee and stingless bee honey may be due to the different pH values of the marinades used. A previous study by Lara et al. [31] indicated that both the values of pH and cooking loss were reduced when antioxidants were added into pork patties. In addition, antioxidants were suggested to confer a protective role against protein denaturation and, thereby, avoid weight loss during the cooking process [18]. Since the honey used in this study was composed of different pH values and antioxidant compounds, it is highly likely that these factors could account for the differences observed in the cooking loss of grilled beef satay marinated with different honey types. 

### 2.3. Internal Temperature

In this study, the internal temperatures (Table 1) of the grilled beef samples ranged from 72.58 to 84.17 °C, thereby indicating that the cooked samples were safe from microbial contamination [32]. The high internal temperature of unmarinated grilled beef was possibly due to the direct contact of beef meat with the flame during the cooking process which consequently increased the cooking loss and resulted in a high internal temperature. However, the lower internal temperatures observed for marinated grilled beef were attributed to the layer of a coating formed from marinades applied before the grilling process which protected the meat from direct contact with the flame and in turn, lowered the internal temperature of the grilled beef. Based on the comparison between internal temperatures of beef meat marinated with table sugar and honey, the internal temperature of beef meat marinated with table sugar had a higher internal temperature than the honey-containing marinades. This observation was due to the high cooking loss during the grilling process of beef meat marinated with table sugar. However, for the samples marinated in stingless bee honey, the lower values of cooking loss were possibly attributed to the ability of the meat to retain water because of the lower pH as compared to the marinade containing honeybee honey. Aktas et al. [33] have studied the effect of organic acid marination on tenderness, cooking loss and bound water content of beef. They found that beef samples with low pH (below pH 5.2) had higher moisture uptake and lower cooking loss. The same result was reported by Medynski et al. [34] in their study.

### 2.4. LOD, LOQ, and Recovery of HCAs

The LOD and LOQ values of HCAs varied from 0.05 to 0.67 and 0.14 to 2.22 ng/g, respectively. The correlation coefficient of each HCA was more than 0.98, thereby indicating a strong association between the peak area and HCA concentration. The recovery of HCAs ranged from 70.25 to 121.37%. In contrast, Zeng et al. [35] reported lower recoveries of HCAs as compared to the results in this study, whereby the values obtained ranged between 53.6 and 108.4%. The differences in the recovery of HCAs may be associated with the different food samples used, low concentrations of mutagens present, and the use of several isolation steps that could hinder the accurate quantification of HCAs [36,37]. The LOD values were in the range of 0.04 and 0.67 ng/g, while the LOQ values were in between 0.14 and 2.22 ng/g.

### 2.5. Amino Acids, Creatinine, and Sugars Profile (Fructose, Glucose, Sucrose, Maltose)

Amino acids, creatinine, and sugars (reducing sugars) are important reactants in the formation of HCAs via the Maillard reaction. The amino acids, creatinine, and sugar content of raw beef (unmarinated) and marinated beef samples prior to the cooking process are presented in Table 2. The unmarinated sample had the lowest total amino acid content of 30.05 µmol/g and was significantly different as compared to the marinated beef samples. The higher amounts of total amino acids found in the marinated samples may be attributed to the types of marinade used. Moreover, it is also likely that the amino acids present in spices and honey used in the marinade may contribute to the increased total amino acid content. The results in this study, however, are in disagreement with a previous study by Hasnol et al. [27], in which the authors reported a lower amino acid content in marinated chicken. 

Creatine or creatinine has an important role in the formation of AIAs as it is responsible for the formation of the imidazo chemical structure in HCAs. Besides, it is also responsible for the mutagenic activity of HCAs as evaluated by the Ames test [38]. In this study, the creatinine levels in raw beef meat samples were 0.04 µmol/g beef meat. This result was lower compared to the creatinine levels reported by Persson et al. [39].

The total sugar content of marinated beef samples ranged from 6.24 µmol/g to 10.32 µmol/g, with significant differences observed among all the beef samples investigated except for beef marinated with starfruit and table sugar. Among the beef samples marinated with stingless bee honey, beef marinated with gelam honey marinade had the highest (10.32 µmol/g) total sugar content, while the beef marinated with acacia honey marinade had the lowest total sugar content at 6.05 µmol/g. This observation could be attributed to the high sugar content in gelam honey as compared to acacia honey. In addition, glucose and fructose were the main sugar constituents detected in all the marinated beef samples, while only glucose was found in the unmarinated samples. Nevertheless, these findings were shown to be consistent with the results obtained by Persson et al. [39]. 

### 2.6. HCAs Content in Grilled Beef Satay

**Figure 1:**
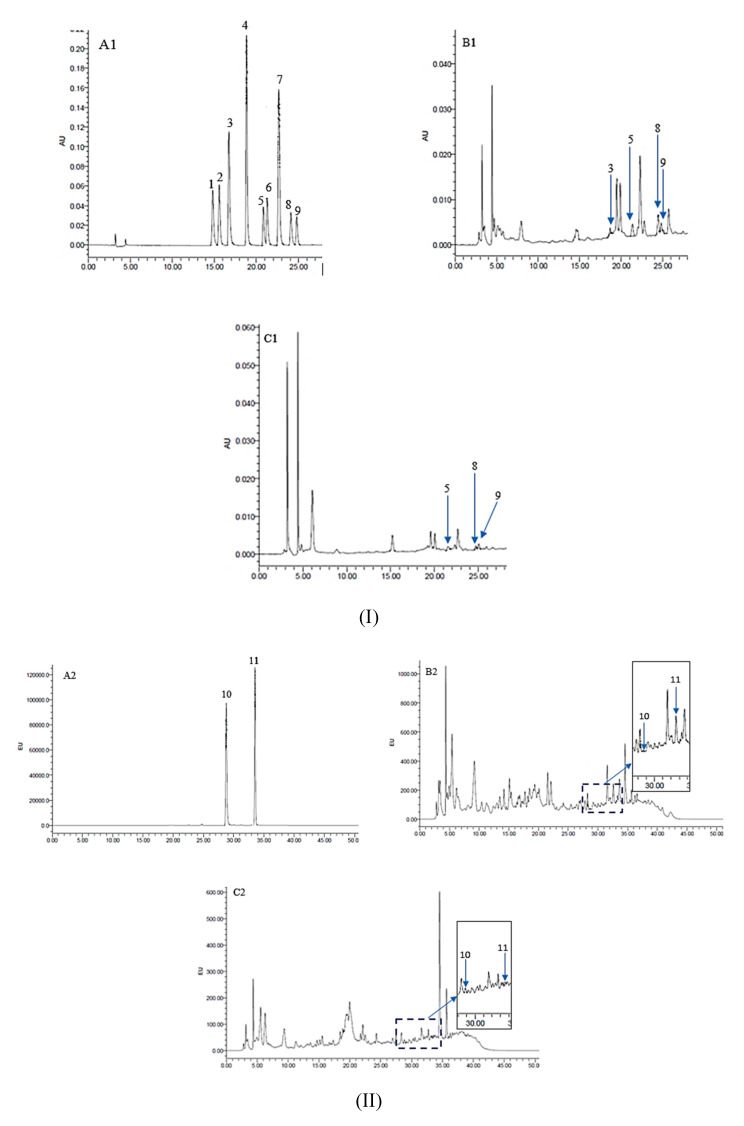
(**I**) HPLC chromatogram of the HCAs standard mix (**A1**) detected by UV detector (λ = 256 nm). HCAs in grilled beef marinated with *Apis* honey (**B1**); HCAs in grilled beef marinated with gelam honey (**C1**). (**II**) HPLC chromatogram of the HCAs standard mix (**A2**) detected by fluorescence detector. HCAs in grilled beef marinated with *Apis* honey (**B2**); HCAs in grilled beef marinated with gelam honey (**C2**). Peaks: 1 = IQ; 2 = IQx; 3 = MeIQ; 4 = MeIQx; 5 = 4,8-DiMeIQx; 6 = 7,8-DiMeIQx; 7 = TriMeIQx (IS); 8 = norharman; 9 = harman; 10 = PhIP and 11 = AαC.

The concentrations of HCAs and its constituent profiles identified in grilled beef satay are shown in Table 3, while the corresponding HPLC chromatograms (samples marinated with *Apis* and gelam honey) are illustrated in Figure 1. In total, ten HCAs were evaluated in this study, but only four to six HCAs were detected in the grilled beef samples, while IQ, IQx, MeIQx, and 7,8-DiMeIQx were not detected in any of the samples. Additionally, it was also observed that the contents of each HCA were greatly varied among the samples. The concentration of HCAs in grilled beef ranged from not detectable to 344.62 ng/g. In all the grilled beef samples, norharman had the highest concentration, ranging from 11.93 ng/g to 314.00 ng/g, followed by harman (3.00 to 14.98 ng/g), MeIQx (not detectable to 5.25 ng/g), AαC (0.08 to 2.00 ng/g), and PhIP (0.06 to 1.22 ng/g). Gross and Gruter [40] also reported similar findings, in which norharman was detected at the highest level in pan-broiled, oven-cooked, and barbecued fish. However, the results in this study contradicted with the findings by Puangsombat et al. [41], whereby the authors showed that PhIP was the most abundant HCA found in chicken, meat, pork, and fish cooked by frying and baking at two different degrees of doneness (medium rare and well done). In a separate study by Jinap et al. [42], no PhIP was detected in chicken and beef satay cooked using different cooking methods such as charcoal-grilled, microwave-charcoal grilled, and microwave-deep frying. Interestingly, DiMeIQx was the most abundant HCA found in grilled beef satay marinated with different types of herbs (turmeric, torch ginger, curry leaf, and lemongrass) and in grilled chicken marinated with different sugars (table sugar, brown sugar, and honey) [30,43]. Therefore, it is anticipated that the differences in the recipes used to prepare the meat, cooking conditions (cooking method, cooking temperature, and cooking time), size/thickness of meat samples, and efficiency of heat transfer could contribute to the inconsistencies in the results observed for HCAs in cooked meat [41]. 

As shown in Table 3, AIAs and amino carbolines compounds were detected in the grilled beef samples. The total content of AIAs (MeIQx, 4,8-DiMeIQx, and PhIP) ranged between 0.81% and 11.27%, while the amino carbolines (norharman, harman and AαC) represented approximately 88.73% to 99.19% of all the HCAs identified. These results were consistent with those reported by Szterk et al. [44], in which higher amino carbolines HCAs compared to AIAs were observed in sirloin and rib steaks grilled at 180 °C for 10 min. In this study, four HCAs were detected in unmarinated beef samples, while additional HCAs were detected in the marinated samples (five to six HCAs). This observation may be justified based on the addition of various ingredients such as spices and herbs to the beef meat during the marination process, thereby adding complexity to the reactions involved in the formation of HCAs. Furthermore, the marinated beef samples were also found to have adequate amounts of free amino acids, reducing sugars, and creatinine which could potentially generate a variety of HCAs. For instance, various spices such as coriander, cumin, and fennel powders were mixed with other herbs and honey types in the marinated beef samples. As previously identified by Zeng et al. [35], cumin and fennel powders can favor the formation of HCAs in cooked meat. Additionally, the results obtained in this study were consistent with a previous study that detected more HCAs in marinated ground mutton [45].

Interestingly, an unexpected result was observed for the unmarinated beef samples, in which the total HCAs in unmarinated beef was shown to be lower (149.76 ng/g) than the samples marinated with table sugar (344.62 ng/g). This observation may be attributed to the significantly lower amounts of amino acids found in unmarinated beef as compared to the beef samples marinated with table sugar. As previously mentioned, amino acids are one of the precursors in the formation of HCAs. Moreover, the different types of precursors and precursor concentrations, as well as molar ratios between these precursors, can influence HCAs generation [9]. Wang et al. [8] reported similar results, in which the total HCAs in marinated samples were found to be higher than the unmarinated samples. However, based on the comparison between samples marinated with table sugar (control) and honey, the total HCAs in samples marinated with honey (*Apis* and stingless bee honey) were observed to be lower than samples marinated with table sugar. This might be due to the lower cooking loss of samples marinated with honey as compared to the samples marinated with table sugar. As reported by Persson et al. [39] in their study, high cooking loss can promote the formation of HCAs in cooked meat.

Norharman and harman derived from β-carboline HCAs were also detected in the grilled beef samples with levels ranging from 11.93 to 314.00 ng/g and 2.43 to 14.99 ng/g, respectively. Norharman was the most abundant form of HCAs identified in all the samples, in which a statistically significant difference was observed among the grilled beef samples from different marinades. Among these marinated samples, beef marinated with table sugar and gelam honey displayed the highest (314.00 ng/g) and the lowest (11.93 ng/g) levels of norharman, respectively. Likewise, Gibis et al. [46] also indicated that norharman was the most abundant form of HCAs identified in fried bacon, while Bordas et al. [47] identified norharman as the highest HCA formed using a meat flavor model system in wet conditions. Additionally, high levels of norharman were also found in broiled beef (795 ng/g), chicken (622 ng/g), and mutton (458 ng/g) [48]. As reported in previous studies, norharman was shown to be generated from the pyrolysis of amino acids, in which tryptophan was identified as the main precursor [49], while glucose was thought to enhance its formation [50]. However, as shown in Table 3, there were no significant differences observed in the tryptophan levels from different marinade samples, thereby indicating that tryptophan was not the key precursor liable for the formation of norharman in this study. Apart from the tryptophan and glucose content, the type of cooking equipment material used can also contribute to the high yield of HCAs [46]. For instance, Ziegenhagen et al. [51] previously reported that the addition of ferric (Fe^2+^) or cupper (Cu^2+^) using a model system resulted in the increase of norharman. Hence, the metal grill bar used in this study could contribute to the high amounts of norharman in grilled beef. 

In addition to norharman, PhIP was also detected in all the samples at lower concentrations as compared to other HCAs, with values ranging from 0.01 to 1.22 ng/g. However, these values were much lower as opposed to the values obtained for grilled chicken (6.74–9.87 ng/g) marinated with different sugar types and cooked at 300 °C [27]. In contrast, no PhIP was detected in chicken and beef satay (medium-cooked) cooked using different cooking methods [42]. In a recent study by Jinap et al. [43], PhIP content ranging from 4.42 to 7.87 ng/g was identified in grilled chicken marinated with different types of sugars and different levels of organic acid concentrations prior to cooking at 300 °C. The difference in the PhIP concentration levels may be attributed to the different types of meat used, cooking times, cooking levels, heat transfer, precursor concentrations, and ingredients used in the marinades. 

The concentrations of AαC in the marinated grilled beef samples, however, were shown to range between not detectable to 1.57 ng/g. Additionally, significant differences were observed between the concentration levels for all samples marinated with different honey types except for the *Apis* honey-containing marinade. Szterk [52] demonstrated that most of the amino acids have a strong association with the formation of different HCAs, while no amino acids have been reported to be involved in the generation of AαC. In an earlier study by Szterk et al. [53], the authors reported that several peptides (carnosine or anserine) in meat could act as precursors and thus, affect the formation of several amines such as Trp-P-1, Trp-P-2, MeAαC, and AαC. 

The concentrations of MeIQx and 4,8-DiMeIQx identified in this study ranged from not detectable to 5.25 ng/g and not detectable to 8.23 ng/g, respectively, in which the highest levels of MeIQx (5.25 ng/g) and 4,8-DiMeIQx (8.23 ng/g) were found in beef marinated with table sugar. On the other hand, the samples marinated with honey had a lower content of MeIQx and 4,8-DiMeIQx, which were not detected in the unmarinated samples. This observation may be due to the low precursor content found in unmarinated samples as compared to marinated samples. Interestingly, MeIQx was not detected in beef marinated with gelam and starfruit honey, although it was present in beef marinated in *Apis* and acacia honey. This observation could be explained by the different inhibitory effects displayed by the different honey-containing marinades. This likely due to the different types of antioxidant compounds present which may exhibit different mechanisms of interactions in relation to the formation of different HCAs [17]. In contrast, Jinap et al. [43] reported higher levels of MeIQx and 4,8-DiMeIQx in grilled chicken marinated with different types of sugars, in which the values ranged from 6.24 to 12.90 ng/g and 10.20 to 56.30 ng/g, respectively. However, in a separate study by Jinap et al. [42], undetectable levels of 4,8-DiMeIQx and 5.18 ng/g of MeIQx was found in charcoal-grilled chicken and beef satay (medium-cooked). 

In this study, however, IQ, IQx, MeIQ, and 7,8-DiMeIQx AIAs were not detected in any of the meat samples analyzed. These results were consistent with Gibis [54], in which no detection of IQ, IQx, MeIQx, and 7,8-DiMeIQx was observed in fried beef marinated with garlic, lemon juice, and onion cooked at 230 °C for 5 min. Likewise, in a different study by Gibis et al. [46], similar observations were also reported.

### 2.7. The Influence of Types of Marinades

The unmarinated samples produced four types of HCAs (norharman, harman, AαC, and PhIP) at low concentrations (0.63–141.64 ng/g), while samples marinated with table sugar produced more types of HCAs (norharman, harman, AαC, PhIP, MeIQx, and 4,8-DiMeIQx) at higher concentrations (0.18–314.00 ng/g). It was suggested that adding spices and sugar in the meat before cooking could favor HCAs formation [45]. Zeng et al. [35] reported that cumin and fennel could promote HCAs formation; these two spices were used in this study as marinade ingredients. Samples marinated with honey produced lower HCAs concentration (0.01–134.16 ng/g) than samples marinated with table sugar (0.18–314 ng/g). This result might be due to the antioxidant activity of the honey as the correlation results showed that there was a strong and significant correlation between total HCAs and antioxidant activity (IC_50_). Previous studies have shown that antioxidant compounds could eliminate free radicals that formed during HCAs formation [22,55].

### 2.8. Reduction of HCAs

To measure the effect of marinades on the reduction of HCAs in grilled beef satay, a comparative analysis was performed between the samples marinated with honey and table sugar, a standard marinade ingredient used in preparing grilled beef satay. In this study, beef marinated with table sugar produced the highest (344.62 ng/g) levels of HCAs during the grilling process. Besides, a significant difference was observed in the production of HCAs between beef samples marinated with table sugar and samples marinated with honey. The total content of HCAs found in beef marinated with *Apis*, acacia, starfruit, and gelam honey were 147.42 ng/g, 49.44 ng/g, 39.79 ng/g, and 16.75 ng/g, respectively, and were found to be significantly lower than beef samples marinated with table sugar, as shown in Table 3. Hence, it is evident that the reduction in HCAs was likely due to the addition of honey in the marinade ingredients. Honey has been reported as a potential source of natural antioxidants and its contents vary according to the botanical sources of honey [24]. It has also been reported in a previous study that antioxidants have an inhibitory effect on the formation of HCAs during the cooking process [56]. Antioxidants potentially act as inhibitors along the different pathways of the reaction, thereby preventing the formation of mutagens in the presence of radical quenchers and free radical scavenger’s activity [22]. In addition, antioxidants can also scavenge free radicals and react with the Maillard reaction products such as the reactive carbonyl species (RCS) arising from thermal and/or Strecker degradation reactions in the formation pathway of HCAs [23,55].

Gibis and Weiss [6] and Quelhas et al. [57] previously reported that antioxidants present in the marinade ingredients could potentially reduce the formation of HCAs in cooked meat. Based on the evaluation of the beef samples marinated in different honey types, all the investigated samples showed a reduction in the formation of HCAs. For instance, the inhibitory effect of gelam, starfruit, acacia, and *Apis* honey marinades was 95.14%, 88.45%, 85.65%, and 57.22%, respectively. Additionally, all these samples showed significant differences in their total HCAs content, thereby indicating that honey obtained from different botanical sources exhibited different inhibitory effects on the formation of HCAs. Clearly, these observations could be attributed to the different types and concentrations of antioxidant compounds present in the honey samples. Among the different honey marinades used, gelam honey showed the highest (95.14%) inhibitory effect on HCAs, possibly because of the high antioxidant activity (IC_50_) observed in gelam honey as compared to other honey types as reported in our previous study [58]. Nevertheless, the results in this study were consistent with the findings by Puangsombat and Smith [59] and Jamali et at. [18], in which the authors demonstrated that the addition of rosemary and tea extracts before cooking reduced the formation of HCAs in beef patties. 

### 2.9. Multivariate Data Analysis

#### 2.9.1. Orthogonal Partial Least Squares Regression

**Figure 2:**
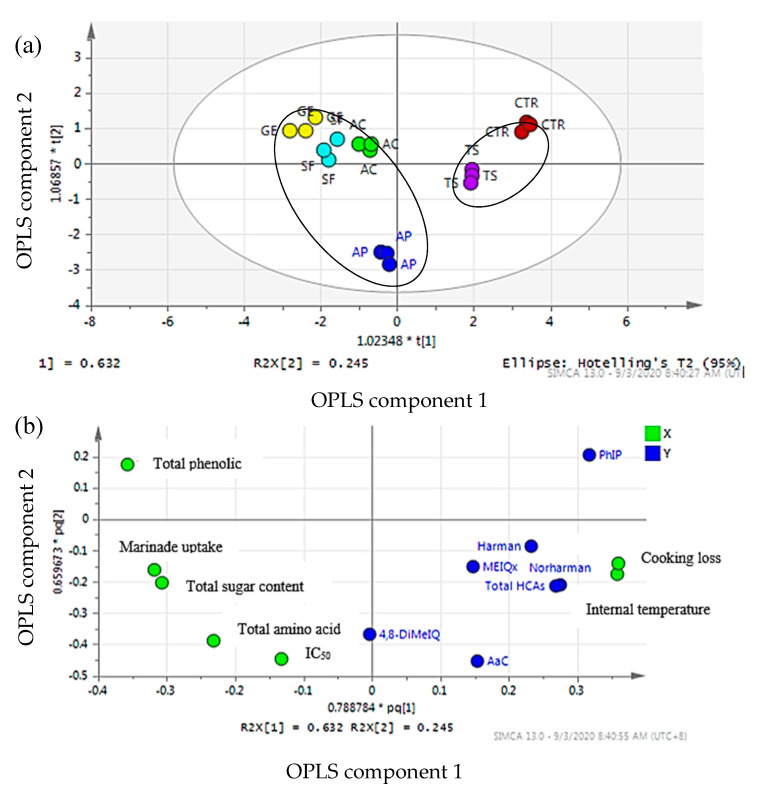
The orthogonal partial least squares (OPLS) score plot (**a**) OPLS loading scatter plot (**b**) of grilled beef (satay) marinated with different marinades. X = marinades uptake, cooking loss, internal temperature, total sugar, total amino acid, and antioxidant properties (total phenolic content (TPC) and antioxidant activity (IC_50_)); Y = types of HCAs and total HCAs. AC = acacia honey; SF = starfruit honey; GE = gelam honey; AP = *Apis* honey; TS = table sugar; CTR = unmarinated.

Orthogonal partial least squares regression (OPLS) was applied to study the effect of different marinades on the formation of HCAs by correlating HCAs concentration formed in grilled beef satay with the marinade uptake, cooking loss, internal temperature, total sugar, total amino acid, and antioxidant properties (total phenolic content (TPC) and antioxidant activity (IC_50_)). Four OPLS components were generated, accounting for 94.70% of the total variance in the data. Based on OPLS component 1 (Figure 2a), a good separation was observed between grilled beef satay samples marinated with honey, samples marinated with table sugar and unmarinated samples. It was suggested that honey-containing marinades had a significant effect on the formation of HCAs in the grilled beef satay. The separation of OPLS component 2 showed that grilled beef marinated with stingless bee honey can be further differentiated from samples marinated with *Apis* honey. This indicated that both stingless bee and *Apis* honey had different effects on HCAs formation. 

To determine the factor that contributes to the discrimination among grilled beef samples, OPLS loading scatter plot was performed (Figure 2b). It was clearly shown that HCAs formation in samples marinated with table sugar and unmarinated samples was strongly correlated with internal temperature and cooking loss. While antioxidant activity (IC_50_), total amino acid, total sugar, marinade uptake, and total phenolic content (TPC) were found to have a higher correlation with the samples marinated with honey.

#### 2.9.2. Partial Least Squares Regression

**Figure 3:**
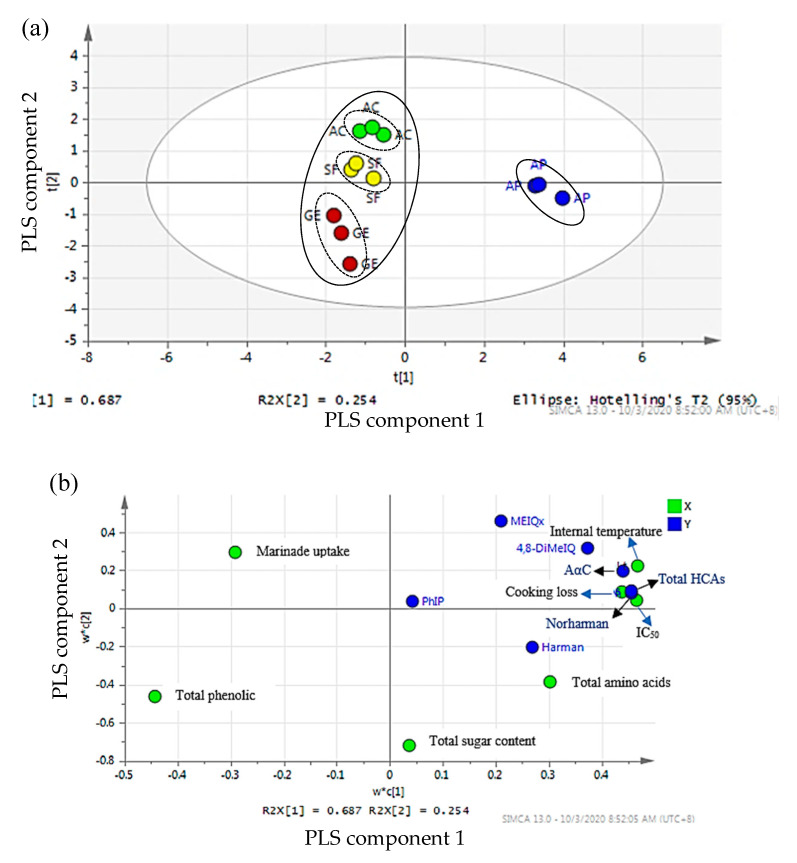
The partial least squares (PLS) score plot (**a**) PLS loading scatter plot (**b**) of grilled beef (satay) marinated with different honey marinades. X = marinades uptake, cooking loss, internal temperature, total sugar, total amino acid, and antioxidant properties (total phenolic content (TPC) and antioxidant activity (IC_50_)); Y = types of HCAs and total HCAs. AC = acacia honey; SF = starfruit honey; GE = gelam honey; AP = *Apis* honey.

To differentiate between samples marinated with stingless bee honey and *Apis* honey, samples marinated with table sugar and unmarinated samples were removed and partial least squares regression (PLS) model was constructed (Figure 3a). Three PLS components were generated, explaining 81.80% of the total variance. The score scatter plot (Figure 3b) shows a complete separation between samples marinated with *Apis* and stingless bee honey. HCAs formation in samples marinated with *Apis* honey had a strong correlation with internal temperature, cooking loss and antioxidant activity (IC_50_) (Figure 3b). This was due to the high internal temperature, cooking loss, and IC_50_ values in grilled beef samples marinated with *Apis* honey than those marinated with stingless bee honey. Honey with high IC_50_ value had low antioxidant activity [58]. This observation clearly showed that *Apis* honey with the high IC_50_ value produced higher HCAs concentration in grilled beef as compared to stingless bee honey, which has lower IC_50_ value. It provided a concrete conclusion that antioxidant compounds contributed to the reduced levels of HCAs in grilled beef marinated with stingless bee honey. Besides, there was some segregation among grilled beef samples marinated with stingless bee honey from different botanical origins (Figure 3a). This might be attributed to the differences in IC_50_ values between them [58].

## 3. Materials and Methods 

### 3.1. Chemicals and Reagents

All HCA standards used were purchased from Toronto Research Chemicals (Toronto, ON, Canada). These standards comprise 2-amino-3-methylimidazo [4,5-f]quinoline (IQ), 2-amino-3-methyl-3*H*-imidazo [4,5-f]quinoxaline (IQx), 2-amino-3,4-dimethylimidazo[4,5-f]quinoline (MeIQ), 2-amino-3,8-dimethylimidazo[4,5-f]quinoxaline (MeIQx), 2-amino-3,4,8-trimethylimidazo[4,5-f]quinoxaline (4,8-DiMeIQx), 2-amino-3,7,8-trimethylimidazo[4,5-f]quinoxaline (7,8-DiMeIQx), 2-amino-1-methyl-6- phenylimidazo[4,5-b]pyridine (PhIP), 2-amino-9*H*-pyrido[2,3-b]indole (AαC), 1-methyl-9*H*-pyrido[3,4-b]indole (Harman), and 9*H*-pyrido[3,4-b]indole (Norharman). Each standard was dissolved in methanol at a concentration of 100 ng/g (stock solution) and used for further dilutions. 2-Amino-3,4,7,8-tetramethyl-3*H*-imidazo[4,5-f] quinoxaline (4,7,8-TriMeIQx) was used as an internal standard (10 ng/g methanolic solution). Acetonitrile, methanol, phosphoric acid (85%), sodium hydroxide, ammonium hydroxide (25%), trimethylamine, and hydrochloric acid were obtained from Sigma and Merck (Darmstadt, Germany). All the chemicals and solvents were of HPLC or analytical grade and water was purified before use (Elga LabWater Ltd., Marlow, UK). 

Diatomaceous earth-based solid-phase columns (Extrelute 20) were obtained from International Sorbent Technology, Hengoed Mid Gleam (UK) and Oasis MCX cartridges (3 cm^3^/60 mg) were purchased from Waters (Milford, MA, USA). A gas-grill stove (auto-ignition) (Clay City, Malaysia), a waring blender (model MX337, Panasonic Corp, Osaka, Japan) and a thermocouple thermometer type-K (Fluke, Everett, WA, USA) were used in this study. 

### 3.2. Raw Materials

Fresh local beef meat (tenderloin) was purchased from a cow farm and all the marinade ingredients were purchased from a wet market at Bandar Baru Bangi, Selangor, Malaysia. Beef meat was stored at −20 °C, while the marinade ingredients were stored at 4 °C prior to marination. All stingless bee honey samples were obtained from three stingless bee farms located in Malacca (gelam honey), Johor (acacia honey) and Pahang (starfruit honey). *Apis mellifera* honey (acacia) from Johor was used as comparison in this study. The samples were subjected to a dehumidification process (40 °C) to reduce the moisture content around 13–15 mg/100g honey and kept at 4 °C prior to use [60,61].

### 3.3. Beef Satay Preparation

Frozen local beef meat (5 kg) was thawed for 24 h in the chiller at 4 °C. The fat was trimmed, and the meat was cut into small cubes (approximately 0.5 cm thick × 2 cm width) prior to washing under running water and draining to remove excess water. The preparation method used in this study was adapted from Jinap et al. [42] and Ahmad Kamal et al. [62] with some modifications. The standard marinade mixtures consisted of shallots (15%), fresh lemongrass (10%), fresh galangal (10%), turmeric powder (5%), cumin powder (5%), fennel powder (5%), coriander powder (10%), table sugar (10%), salt (1%), and cooking palm oil (1%). In treated marinade mixtures, honey was used to replace the table sugar in the standard marinade mixtures. Stingless bee honey derived from gelam (13.50%), acacia (13.90%) and starfruit (14.10%) as well as honeybee honey (*Apis mellifera*) (14.50%) were separately added into the standard marinade mixtures based on the calculated relative total sugar content described by Hasnol et al. [27]. All the herbs (fresh lemongrass, fresh galangal, and shallots) were blended using a waring blender for 5 min before mixing with other ingredients (cumin, fennel, turmeric powder, sugar or honey, salt, and cooking oil). The beef meat (100 g) was put into a polyethylene plastic bag, mixed with the marinade mixture by hand, and marinated for 24 h in a chiller at 4 °C. The marinated raw beef cubes were skewed onto bamboo skewers and grilled using a gas-grill stove. The experiment was performed three times. For unmarinated beef samples, no marinade mixture was added.

### 3.4. Grilling Conditions

All skewed beef satay samples marinated with different stingless bee honey-containing marinades were cooked using a gas-grill stove. The grill bar was pre-heated, and the temperature of the grill bar was measured using a thermocouple thermometer probe (A type-K, Fluke Corporation, Everett, WA, USA) clipped to the grill bar. When the surface temperature reached 265 ± 5 °C, three beef satay skewers were cooked for 3.5 min on each side. The internal temperature of each beef satay was measured after the grilling process using the thermocouple thermometer (A type-K, Fluke Corporation, Everett, WA, USA). Unmarinated beef cube samples were also treated under the same grilling conditions. These grilling experiments were conducted for each honey-containing marinade and replicated three times. No salt or oil was applied to the beef satay samples during or after the grilling process [30]. 

### 3.5. Marinade Uptake

The weight gain for each beef satay sample was measured by subtracting the sample weight obtained before and after the marinating process as previously described by Jinap et al. [30].

### 3.6. Cooking Loss

The cooking loss of each beef satay sample was calculated after the grilling process. The cooked beef satays were left to reach room temperature for approximately 30 min and the cooking loss was determined based on the method described by Puangsombat et al. [41]. The following formula was used: Cooking loss = (A − B)/A × 100%. Where A is the weight of marinated beef and B is the weight of cooked beef.

### 3.7. Analysis of HCAs

#### 3.7.1. HCAs Extraction and Clean-Up Procedures

The extraction and purification of HCAs of beef satay samples were performed using a solid-phase extraction method developed by Gross and Gruter [40] and modified by Messner and Murkovic [63] with slight amendments. Ground beef satay samples (2 g) were mixed with 20 mL of 1 M NaOH and homogenized for 2 h using a magnetic stirrer at 1000 rpm. The samples were mixed with 15 g of Extrelut refill material (diatomaceous earth) as the stationary phase and placed in a 20 mL Extrelut column. Ethyl acetate (50 mL) was used as the extraction solvent in this study. The extracted sample (25 mL) was passed through the Oasis MCX cartridge (3 cm^3^/60 mg) (Waters, Milford, MA, USA) which was pre-conditioned with 2 mL of ethyl acetate. The column was washed using 2 mL of 0.1 M HCl and followed by 2 mL of methanol. The HCAs compounds were subsequently eluted with 2 mL of methanol in concentrated ammonia (19:1, *v/v*). The HCAs fraction was evaporated to dryness under a stream of nitrogen at 50 °C. The final HCAs extract was dissolved in 100 μL of methanol [27].

#### 3.7.2. HPLC Analysis of HCAs

The determination of HCAs was performed according to the modified method described by Quelhas et al. [57]. A high-performance liquid chromatography (HPLC) (Waters 600, Milford, MA, USA) instrument equipped with a photodiode array detector (PDA) (Waters 2996, Milford, MA, USA) and fluorescence (Waters 2475, Milford, MA, USA) detector was used in this study. The PDA detector was used to detect IQ, IQx, MeIQ, MeIQx, 7,8-DiMeIQx, 4,8-DiMeIQx, TriMeIQx (internal standard), Norharman, and Harman at a wavelength of 256 nm. On the other hand, the fluorescence detector was used to detect PhIP and AαC at an excitation/emission wavelength of 307/370 nm. A reverse-phase TSK-gel ODS 80-TM column (5 µm particle size, 4.6 mm × 250 mm) from Tosoh Bioscience GmBH (Stuttgart, Germany) was used to isolate the HCAs compounds. Approximately 20 µL of the sample was injected into the HPLC and separation was performed with acetonitrile (A), 0.01 M trymethylamine, pH 3.2 (B), and 0.01M trymethylamine, pH 3.6 (C) at a flow rate of 1.0 mL/min using gradient elution: 0–10 min, 5–15% A, and 95% B; 10.1–20 min, 15% A, and 85% C; 20.1–30 min, 25% A, and 75% C; 30.1–33 min, 55% A, and 45% C; 33.1–34 min, 5% A, and 95% B and continued for 55 min. The total analysis run time was 55 min [40]. The identification of HCAs was carried out by comparing the retention time of unknown peaks in the sample chromatogram with reference standards. 

#### 3.7.3. LOD, LOQ, and Recovery of HCAs

Pure standards consisting of ten HCAs were dissolved in methanol to produce individual pure standard solutions of 10 ppm (IQx) and 100 ppm (IQ, MeIQ, MeIQx, 7,8-DiMeIQx, 4,8-DiMeIQx, Norharman, Harman, PhIP, and AαC), respectively. TriMeIQx (10 ppm) was used as an internal standard. Subsequently, a mixed standards solution was prepared by mixing different volumes of each pure standard solution of HCAs. The final concentrations of HCAs in the standards solution mixture were as follows: 1.04 ppm (4,8-DiMeIQx and Harman), 1.31 ppm (7,8-DiMeIQx), 2.61 ppm (IQ), 3.13 ppm (IQx), 5.22 ppm (MeIQ), 7.83 ppm (MeIQx and PhIP), 10.44 ppm (Aαc), 13.05 ppm (Norharman), and 18.28 ppm (TriMeIQx). Five serial dilutions of mixed standards were prepared by adding different volumes of methanol with 50 µL of mixed standards solution. The serial dilutions and corresponding peak areas were used to construct a standard curve for each HCAs compound [64]. The coefficients of determination (*r*^2^) for the standard curves were greater than 0.98 for all the HCAs identified in this study.

The limits of detection (LOD) and limits of quantification (LOQ) for each standard were determined using the regression line method. LOD and LOQ were calculated as 3 *sd/slope and 10 *sd/slope, respectively [65]. The recovery rates for different HCAs were determined by the standard edition method. The blank samples (raw beef meat) were spiked with three different concentrations of HCA mixture standards and the recovery rate was calculated by comparing the concentration of the fortified samples with those of the unfortified samples [42]. 

### 3.8. Determination of Precursors in Raw Beef Satay Samples

#### 3.8.1. Amino Acids

Amino acids were determined according to the method described by Perez-Palacios et al. [66] with slight modifications. Homogenized raw beef satay samples (2 g) were suspended in 15 mL of 0.10 M HCl and stirred using a magnetic stirrer at 1000 rpm for 5 min. Approximately 2 mL of supernatant was subjected to centrifugation (Refrigerated Centrifuge 3-18K, Sigma, Gilingham Dorset, UK) at 10,000 rpm or 10,956× *g* for 15 min at 4 °C. Subsequently, 100 µL of supernatant was mixed with 250 µL acetonitrile to deproteinize the sample and the solution was centrifuged at 10,000 rpm for 3 min. Approximately 100 µL of the supernatant was subjected to the derivatization process. The samples were further analyzed according to the manufacturer’s instructions provided in the EZ:faast^TM^ GC-MS free amino acid analysis kit obtained from Phenomenex Inc. (Torrance, CA, USA) [67]. Data from the analysis were recorded in triplicates. For the honey samples, a volume of 0.10 g of pure honey was dissolved in 500 µL of distilled water, vortexed for 1 min and approximately 30 µL of the honey solution was subjected to the derivatization process [67].

#### 3.8.2. Sugars Profile (Fructose, Glucose, Maltose, and Sucrose)

Fructose, glucose, maltose, and sucrose were determined using HPLC according to the method described by Hasnol et al. [27]. Briefly, raw beef satay samples (1 g) were homogenized in 10 mL of acetonitrile:water (50:50, *v/v*) for 2 h. The suspension was subjected to centrifugation (Refrigerated Centrifuge 3-18K, Sigma, Gilingham Dorset, UK) at 5000 rpm or 2739× *g* for 20 min at 4 °C. The supernatant was filtered through a 0.45 µm nylon syringe filter (Whatman, Buckinghamshire, UK) prior to analysis. An aliquot of 20 µL of the filtrate was injected into the Waters HPLC system equipped with a refractive index (RI) detector. The separation was performed using a SUPELCOSILTM LC-NH2 column, 5 µm (250 × 4.6 mm) (Supelco, Bellefonte, PA, USA). Isocratic gradient elution with a mobile phase consisting of acetonitrile:water (80:20, *v/v*) was performed at a flow rate of 1.0 mL/min. The separated peaks were identified based on the comparison with retention times obtained from the sugar standards. The analysis was performed in triplicates.

#### 3.8.3. Creatinine

The creatinine content in raw beef satay was determined using the creatinine kit purchased from Sigma-Aldrich. The extraction, purification, and analysis methods were carried out according to the manufacturer’s instructions provided in the kit. Approximately 0.1 g of raw beef satay sample was added to 400 µL of creatinine buffer assay and subjected to centrifugation at 11,000 rpm or 13,257× *g* for 10 min at 4 °C. Approximately 4 mL of the supernatant was removed from the homogenate, transferred into Amicon^®^ Ultra-4 centrifugal filters (10,000 MWL) (Merck, Darmstadt, Germany), and further centrifuged at 13,000 rpm or 18,515× *g* for 10 min. An aliquot (40 µL) of the supernatant was transferred into an ELISA 96-well plate and the creatinine test was completed according to the manufacturer’s protocol supplied in the creatinine colorimetric assay kit. The creatinine concentrations were measured enzymatically using the creatinine colorimetric/fluorometric assay kit (BioVision Inc., Milpitas, CA, USA). The analysis was carried out in triplicates [62].

### 3.9. Statistical Analysis

The data for pH, total phenolic content (TPC), antioxidant activity (IC_50_) of the honey samples were acquired from our previous study [58] as the same honey samples were used to prepare grilled beef satay. The data obtained were analyzed using the Minitab statistical package Version 16 (Minitab LLC, State College, PA, USA). The values obtained for the statistical analyses were expressed as mean ± standard deviation (SD). A one-way analysis of variance (ANOVA) was performed and the evaluation of the significant differences between the mean values of HCAs for different treatments was performed using the Fisher’s multiple comparison test at a significance level of *p* < 0.05. As for multivariate data analysis, the data were fed to the SIMCA P+14.0 software (Version 14.0, Umetrics, Umea, Vasterbotten, Sweden) and the UV scaling method was employed. Orthogonal partial least squares regression (OPLS) and partial least squares regression(PLS) were developed to determine the relationship between the grilled beef samples marinated with different honey containing marinades and factors responsible for the HCAs formation (total sugar, total amino acid, antioxidant properties, marinade uptake, cooking loss, and internal temperatures) and total HCAs.

## 4. Conclusions

The results from this study have shown that the addition of honey in the marinade mixture is an effective approach to reducing the formation of HCAs in grilled beef during the cooking process. This finding should be of interest to the food manufacturing and food service as a simple and practical method of achieving significant reductions of specific HCAs in grilled beef. Further study needs to be carried out for determining which compound in honey is responsible for suppressing HCAs formation.

## Figures and Tables

**Table 1:** Marinade uptake, cooking loss, and internal temperatures of grilled beef satay.

Treatment	Marinade Uptake (%)	Cooking Loss (%)	Internal Temperature (°C)
Unmarinated	NA	35.38 ± 0.34 ^a^	84.17 ± 0.15 ^a^
Marinated with:			
Table sugar (control)	26.21 ± 0.18 ^c^	34.10 ± 0.25 ^b^	82.47 ± 0.47 ^b^
*Apis mellifera* honey	29.78 ± 0.37 ^b^	32.69 ± 0.66 ^c^	80.83 ± 0.68 ^c^
Acacia honey	33.32 ± 0.43 ^a^	28.88 ± 0.42 ^d^	75.40 ± 0.78 ^d^
Starfruit honey	32.28 ± 0.57 ^a^	28.10 ± 0.29 ^d^	74.03 ± 0.57 ^d,e^
Gelam honey	31.12 ± 0.76 ^a^	28.46 ± 0.39 ^d^	73.00 ± 0.20 ^e^

NA = Not available; Mean values with different lowercase letters in the same row indicate significant differences (*p* < 0.05) between beef meat satays marinated with different marinades.

**Table 2:** Amino acids, creatinine, and sugar content (µmol/g) in marinated beef based on the different marinades.

Amino Acids	Unmarinated Samples	Types of Marination				
		Table Sugar	*Apis Mellifera*	Acacia	Starfruit	Gelam
Alanine	5.51 ± 0.33 ^a^	4.81 ± 0.13 ^b^	5.73 ± 0.22 ^a^	4.57 ± 0.17 ^b^	4.89 ± 0.60 ^b^	4.86 ± 0.12 ^b^
Sarcosine	<LOQ	<LOQ	<LOQ	<LOQ	<LOQ	<LOQ
Glycine	1.96 ± 0.06 ^a^	1.54 ± 0.06 ^b^	1.77 ± 0.20 ^a^	1.24 ± 0.06 ^c^	1.31 ± 0.16 ^c^	1.43 ± 0.05 ^b,c^
α-Amino butyric acid	<LOQ ^c^	<LOQ ^c^	0.19 ± 0.06 ^a^	<LOQ ^c^	0.12 ± 0.00 ^b^	0.16 ± 0.07 ^a,b^
Valine	<LOQ ^d^	1.01 ± 0.09 ^b^	1.29 ± 0.03 ^a^	0.70 ± 0.07 ^c^	<LOQ ^d^	0.80 ± 0.20 ^c^
β-Amino iso butyric acid	2.40 ± 1.06 ^a^	1.77 ± 0.30 ^a^	1.99 ± 0.53 ^a^	1.53 ± 0.63 ^a^	1.94 ± 0.10 ^a^	1.73 ± 0.30 ^a^
Leucine	1.58 ± 0.91 ^a^	1.59 ± 0.50 ^a^	1.37 ± 0.80 ^a,b^	1.85 ± 0.34 ^a^	0.37 ± 0.01 ^b^	1.40 ± 0.89 ^a,b^
Allo-isoleucine	1.21 ± 0.70 ^a,b^	1.50 ± 1.01 ^a,b^	1.92 ± 1.27 ^a^	<LOQ ^b^	2.65 ± 0.22 ^a^	1.31 ± 1.46 ^a,b^
Isoleucine	0.17 ± 0.05 ^c^	<LOQ ^d^	0.67 ± 0.11 ^a^	0.44 ± 0.03 ^b^	<LOQ ^d^	<LOQ ^d^
Threonine	<LOQ ^d^	0.71 ± 0.06 ^b^	0.89 ± 0.08 ^a^	0.50 ± 0.05 ^c^	0.44 ± 0.11 ^c^	0.67 ± 0.05 ^b^
Serine	1.47 ± 0.25 ^d^	2.72 ± 0.10 ^a,b,c^	3.33 ± 0.22 ^a^	2.51 ± 0.01 ^b,c^	3.07 ± 0.29 ^a,b^	2.20 ± 0.92 ^c,d^
Proline	0.33 ± 0.11 ^e^	1.44 ± 0.09 ^c^	2.30 ± 0.15 ^a^	1.18 ± 0.04 ^d^	1.29 ± 0.07 ^c,d^	1.66 ± 0.11 ^b^
Asparagine	<LOQ ^d^	1.56 ± 0.21 ^c^	2.42 ± 0.25 ^b^	1.48 ± 0.30 ^c^	2.06 ± 0.26 ^b^	2.94 ± 0.26 ^a^
Aspartic acid		0.95 ± 0.06 ^b^	2.40 ± 1.50 ^a^	2.47 ± 0.25 ^a^	1.09 ± 0.02 ^b^	1.38 ± 0.71 ^a,b^
Methionine	1.32 ± 0.14 ^b^	1.61 ± 0.02 ^b^	1.64 ± 0.05 ^b^	1.43 ± 0.07 ^b^	2.17 ± 0.19 ^a^	1.52 ± 0.39 ^b^
3-hydroxyproline	<LOQ	<LOQ	<LOQ	<LOQ	<LOQ	<LOQ
Glutamic Acid	1.60 ± 0.56 ^c^	4.50 ± 0.18 ^a^	5.09 ± 0.58 ^a^	3.20 ± 0.35 ^b^	3.52 ± 0.36 ^b^	3.42 ± 0.15 ^b^
Phenylalanine	0.53 ± 0.13 ^d^	1.32 ± 0.05 ^c^	1.39 ± 0.14b ^c^	1.10 ± 0.04 ^c^	2.70 ± 0.34 ^a^	1.76 ± 0.34 ^b^
α-Amino adipic Acid	1.03 ± 0.27 ^a^	0.68 ± 0.14 ^b^	0.72 ± 0.11 ^b^	<LOQ ^d^	0.27 ± 0.08 ^c^	0.47 ± 0.14 ^b,c^
Glutamine	4.28 ± 1.45 ^c^	5.63 ± 0.48 ^b,c^	8.01 ± 0.70 ^a^	6.63 ± 0.32 ^a,b^	7.26 ± 0.84 ^a^	7.95 ± 0.29 ^a^
Ornithine	1.31 ± 0.05 ^b^	2.08 ± 0.22 ^a^	2.33 ± 0.33 ^a^	1.05 ± 0.03 ^b^	1.04 ± 0.22 ^b^	1.08 ± 0.0 ^b^
Lysine	1.27 ± 0.05	2.02 ± 012 ^b^	2.09 ± 0.02 ^a,b^	2.17 ± 0.02 ^a,b^	2.26 ± 0.17 ^a^	2.24 ± 0.08 ^a^
Histidine	1.64 ± 0.04 ^b,c^	1.82 ± 0.01 ^a,b,c^	1.92 ± 0.08 ^a^	1.75 ± 0.14 ^a,b,c^	1.62 ± 0.30 ^c^	1.89 ± 0.12 ^a,b^
Tyrosine	0.70 ± 0.01 ^c^	0.82 ± 0.06 ^b,c^	0.69 ± 0.15 ^c^	0.94 ± 0.01 ^a,b^	0.81 ± 0.18 ^b,c^	1.03 ± 0.10 ^a^
Tryptophan	0.98 ± 0.01 ^a^	1.07 ± 0.004 ^a^	1.07 ± 0.01 ^a^	1.03 ± 0.01 ^a^	1.12 ± 0.25 ^a^	1.05 ± 0.05 ^a^
Cysteine	ND	ND	ND	ND	ND	ND
TOTAL	30.05 ± 1.12 ^d^	41.17 ± 1.15 ^b^	51.22 ± 2.31 ^a^	38.18 ± 0.84 ^c^	42.01 ± 1.50 ^b^	42.96 ± 2.35 ^b^
Creatinine	0.04 ± 0.004					
Fructose	ND ^e^	2.83 ± 0.01 ^d^	4.27 ± 0.12 ^b^	2.96 ± 0.04 ^c,d^	3.20 ± 0.01 ^c^	5.16 ± 0.40 ^a^
Glucose	4.26 ± 0.03 ^b^	3.22 ± 0.02 ^c^	5.33 ± 0.32 ^a^	3.28 ± 0.04 ^c^	4.51 ± 0.06 ^b^	5.16 ± 0.12 ^a^
Sucrose	ND	ND	ND	ND	ND	ND
Maltose	ND	ND	ND	ND	ND	ND
Total sugar	4.26 ± 0.03 ^e^	6.05 ± 0.01 ^d^	9.61 ± 0.44 ^b^	6.24 ± 0.07 ^d^	7.71 ± 0.06 ^c^	10.32 ± 0.52 ^a^

ND = not detected; <LOQ = below limit of quantitation; mean values with different lowercase letters in the same row indicate significant differences (*p* < 0.05) between the marinated beef from different marinades.

**Table 3:** Effect of honey marinades on the formation of HCAs in grilled beef satay (ng/g).

Treatments	Aminoimidazoazaarens (AIAs)			Amino Carbolines			
	MeIQx	4,8-DiMeIQx	PhIP	NH	H	AαC	Total HCAs
CTR	ND	ND	1.22 ± 0.01 ^a^	141.64 ± 1.43 ^b^	6.27 ± 0.28 ^b^	0.63 ± 0.06 ^c^	149.76 ± 1.63 ^b^
TS	5.25 ± 0.32 ^a^	8.23 ± 0.57 ^a^	0.18 ± 0.15 ^b^	314.00 ± 1.57 ^a^	14.99 ± 0.94 ^a^	1.97 ± 0.02 ^a^	344.62 ± 2.77 ^a^
AP	1.29 ± 0.08 ^b^	5.93 ± 0.13 ^b^	0.06 ± 0.07 ^b,c^	134.16 ± 1.80 ^c^	3.98 ± 0.41 ^c^	1.57 ± 0.03 ^a^	147.42 ± 1.59 ^b^
AC	1.78 ± 0.81 ^b^	3.72 ± 0.67 ^c^	0.07 ± 0.05 ^b,c^	40.60 ± 0.86 ^d^	2.43 ± 0.15 ^e^	0.84 ± 0.23 ^b^	49.44 ± 1.72 ^c^
SF	ND ^c^	4.00 ± 0.95 ^c^	0.01 ± 0.01 ^b,c^	31.55 ± 0.58 ^e^	3.69 ± 0.02 ^c,d^	0.53 ± 0.02 ^c^	39.79 ± 1.31 ^d^
GE	ND ^c^	1.68 ± 0.40 ^d^	0.06 ± 0.04 ^c^	11.93 ± 0.27 ^f^	3.00 ± 0.12 ^d,e^	0.08 ± 0.01 ^d^	16.75 ± 0.21 ^e^

ND = not detected; NH = norharman; H = harman; HCAs = heterocyclic amines; mean values with different lowercase letters in the same row indicate significant differences (*p* < 0.05) between grilled beef (satay) from different marinades. CTR = unmarinated sample; TS = table sugar; AP = *Apis* honey; AC = acacia honey; SF = starfruit honey; GE = gelam honey.
